# *Neisseria meningitidis* Factor H Binding Protein Surface Exposure on *Salmonella* Typhimurium GMMA Is Critical to Induce an Effective Immune Response against Both Diseases

**DOI:** 10.3390/pathogens10060726

**Published:** 2021-06-09

**Authors:** Francesca Necchi, Giuseppe Stefanetti, Renzo Alfini, Elena Palmieri, Martina Carducci, Roberta Di Benedetto, Fabiola Schiavo, Maria Grazia Aruta, Fabiola Giusti, Ilaria Ferlenghi, Yun Shan Goh, Simona Rondini, Francesca Micoli

**Affiliations:** 1 GSK Vaccines Institute for Global Health, 53100 Siena, Italy; Giuseppe_Stefanetti@hms.harvard.edu (G.S.); renzo.x.alfini@gsk.com (R.A.); elena.x.palmieri@gsk.com (E.P.); martina.x.carducci@gsk.com (M.C.); roberta.x.di-benedetto@gsk.com (R.D.B.); fabiola.schiavo@yahoo.it (F.S.); maria-grazia.x.aruta@gsk.com (M.G.A.); yunshan.goh@gmail.com (Y.S.G.); simona.x.rondini@gsk.com (S.R.); francesca.x.micoli@gsk.com (F.M.); 2 Department of Immunology, Blavatnik Institute, Harvard Medical School, Boston, MA 02115, USA; 3 Luigi Sacco Department of Biomedical and Clinical Sciences, University of Milan, 20122 Milano, Italy; 4 GSK, 53100 Siena, Italy; fabiola.x.giusti@gsk.com (F.G.); ilaria.x.ferlenghi@gsk.com (I.F.); 5 University of Siena, 53100 Siena, Italy; 6 Agency for Science, Technology and Research (A*STAR) Infectious Diseases Labs, Singapore 138648, Singapore

**Keywords:** GMMA, Salmonella, Neisseria meningitidis, fHbp

## Abstract

GMMA, outer membrane vesicles resulting from hyperblebbing mutated bacterial strains, are a versatile vaccine platform for displaying both homologous and heterologous antigens. Periplasmic expression is a popular technique for protein expression in the lumen of the blebs. However, the ability of internalized antigens to induce antibody responses has not been extensively investigated. Herein, the *Neisseria meningitidis* factor H binding protein (fHbp) was heterologously expressed in the lumen of O-antigen positive (OAg+) and O-antigen negative (OAg−) *Salmonella* Typhimurium GMMA. Only the OAg− GMMA induced an anti-fHbp IgG response in mice if formulated on Alum, although it was weak and much lower compared to the recombinant fHbp. The OAg− GMMA on Alum showed partial instability, with possible exposure of fHbp to the immune system. When we chemically conjugated fHbp to the surface of both OAg+ and OAg− GMMA, these constructs induced a stronger functional response compared to the fHbp immunization alone. Moreover, the OAg+ GMMA construct elicited a strong response against both the target antigens (fHbp and OAg), with no immune interference observed. This result suggests that antigen localization on GMMA surface can play a critical role in the induction of an effective immune response and can encourage the development of GMMA based vaccines delivering key protective antigens on their surface.

## 1. Introduction

Native outer membrane vesicles (nOMVs) are small bi-layered membrane structures naturally released from Gram-negative bacteria [1]. Among many functions, nOMVs allow bacteria to interact with their environment, promote pathogenesis, survive stress conditions, modulate host-pathogen interactions and divert host immune responses [2,3,4]. 

OMVs have been increasingly used in vaccinology, as they contain the antigenic components and the bacterial outer membrane constituents to elicit a protective immune response [4]. In the beginning, detergent extracted outer membrane vesicles (dOMV) were employed in meningitis vaccines [5]. The action of the detergent allowed the depletion of endotoxic lipoproteins and lipooligosaccharides [6,7]. However, dOMV possess a different protein composition from nOMV, and not surprisingly, the latter have shown to induce stronger antibody responses, with increased bactericidal activity, and to elicit a broader range of antibody isotypes [8]. The main limitation of the nOMV approach is the low yield of vesicles released by Gram-negative bacteria. To overcome this issue, genetic methods have been developed to increase the vesicle blebbing [9,10]. The nOMVs resulting from hyperblebbing mutated strains are called GMMA-Generalized Modules of Membrane Antigens [10]. To reduce the endotoxicity of the GMMA, lipid A are genetically altered by different mechanisms, still maintaining the immunogenicity of vaccine preparations [4,6,11,12,13,14]. The most advanced GMMA-based vaccine is against Shigella sonnei [9], which showed good tolerability and immunogenicity in European and Kenyan adults [15,16] and was able to induce a strong anamnestic response after boosting [17]. 

Extracellular vesicles are gaining increasing attention as a vaccine platform, as they can be further manipulated in their protein or polysaccharide content by genetic engineering to overexpress key homologous antigens, or to express heterologous antigens [4]. The display of protein vaccine targets to the bacterial cell surface via genetic manipulation can be achieved either by periplasmic expression, or by fusing the target protein to membrane-associated proteins, such as β-barrel domain of autotransporters, toxins and outer membrane proteins [18,19,20,21,22]. Kesty and Kuehn demonstrated the possibility of manipulating the membrane and periplasmic protein content of blebs produced by laboratory and pathogenic *E. coli* strains to include heterologous proteins [2]. Periplasmic expression is relatively simple and leads to the accumulation of recombinant antigens in the lumen of the blebs, but the ability of internalized antigens to induce antigen-specific antibody responses has not yet been fully investigated [23,24,25]. 

More recently, chemical conjugation has been proposed as a possible tool to decorate GMMA with multiple heterologous proteins or polysaccharide antigens [26].

The expression of heterologous antigens in outer membrane vesicles can be an effective way to tackle multiple diseases in a single vaccine. Our study investigates the co-expression of *Neisseria* and *Salmonella* antigens, with the final aim to cover both non-typhoidal *Salmonella* and *Neisseria meningitidis* diseases prevalent in sub-Saharan Africa [14,27,28]. The *Neisseria meningitidis* factor H binding protein (fHbp) [29] was expressed and incorporated in the lumen of *Salmonella* Typhimurium GMMA or chemically conjugated to GMMA surface, to investigate the role that antigen exposure can have on induced antibody response. GMMA with and without O-antigen (OAg+ and OAg−) were compared to investigate possible immune interference of *Salmonella* OAg, key antigen for *S.* Typhimurium, on the immune response to fHbp. A panel of analytical methods (Transmission Electron Microscopy (TEM), Fluorescence-Activated Cell Sorting (FACS), competitive ELISA) was used to confirm fHbp localization in GMMA constructs.

Our work investigates the role that antigen localization in GMMA can play to induce an effective immune response and gives insight into the optimal design of GMMA based vaccines covering multiple diseases, suggesting a critical role for surface exposure of the immunogens to stimulate a good humoral response.

## 2. Results

### 2.1. Meningococcal fHbp Expressed in S. Typhimurium Is Incorporated in GMMA with Minimal Alteration of Their Structural Features

*N. meningitidis* fHbp, an outer membrane lipoprotein, was cloned in a pGEX-derived plasmid under an IPTG inducible promoter. The generated plasmid was used to transform the overblebbing *S.* Typhimurium strains 1418 *ΔtolR* (OAg+) and 1418 *ΔtolR ΔwbaP* (OAg−). GMMA were purified from the culture supernatants of the derived strains after inducing antigen expression with IPTG. 

**Figure 1:**
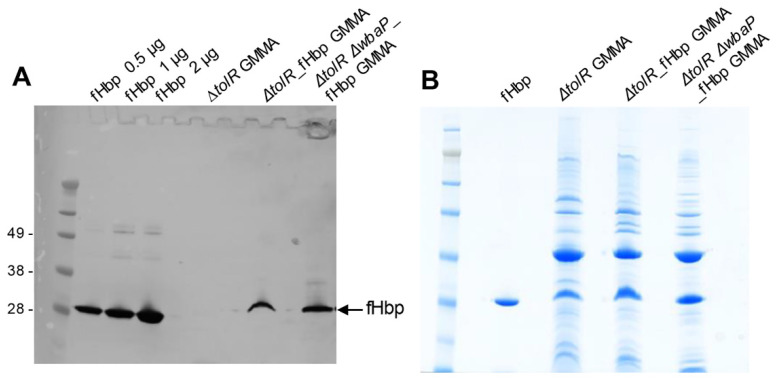
Expression of meningococcal fHbp in *S.* Typhimurium GMMA. (**A**) Western blot analysis by using anti-fHbp mice serum. 10 µg of total protein for GMMA derived from *S.* Typhimurium 1418 *ΔtolR*, 1418 *ΔtolR*_fHbp and 1418 *ΔtolR ΔwbaP*_fHbp strains were loaded. (**B**) SDS-Page analysis of recombinant fHbp (loaded 2 µg) and purified GMMA from *S.* Typhimurium 1418 *ΔtolR*, 1418 *ΔtolR*_fHbp and 1418 *ΔtolR ΔwbaP*_fHbp strains (loaded 15 µg total protein).

A panel of analytical methods was used to characterize the GMMA produced. Highly pure and intact GMMA were obtained and observed by HPLC-SEC analysis, which also revealed the absence of soluble proteins and DNA (data not shown). SDS-Page analysis followed by Western blotting with anti-fHbp serum antibodies showed positive expression of the heterologous antigen in GMMA, for both *S.* Typhimurium 1418 ΔtolR and 1418 *ΔtolR ΔwbaP* strains (Figure 1A). The GMMA protein pattern visualized by SDS-Page was also similar between the two mutant strains expressing fHbp (Figure 1B). FASP/H3 Mass Spectrometry revealed 3.9% of fHbp over total protein content expressed in 1418 *ΔtolR ΔwbaP* GMMA (75% total protein recovery) and 2.2% of fHbp over total protein content for 1418 *ΔtolR* GMMA (65% total protein recovery). 

The particle size of GMMA from the mutant strains expressing fHbp was similar to the respective parent strain, as estimated by both HPLC-SEC analysis coupled with MALS detector and dls (dynamic light scattering) (Table 1). GMMA from 1418 *ΔtolR ΔwbaP* were slightly smaller compared to GMMA from 1418 *ΔtolR* (Table 1). 

*S.* Typhimurium OAg (part of *Salmonella* LPS) is the other key target antigen of our GMMA preparations. Therefore, sugar content was investigated and characterized in all OAg positive GMMA constructs in order to examine how heterologous protein expression can impact OAg expression level and structural features. The w/w ratio of OAg chains per mg of total protein remained high for 1418 *ΔtolR* GMMA after fHbp expression compared to the parent strain (0.9 and 0.66 respectively). LPS content over total protein was also comparable between 1418 *ΔtolR* and *ΔtolR*_fHbp GMMA (Table 1). OAg populations derived from 1418 *ΔtolR* and *ΔtolR*_fHbp GMMA revealed similar sugar composition by HPAEC-PAD, except for a decrease in glycosylation levels after fHbp expression (Table 1). Furthermore, the two main OAg populations with different average MW, consisting of 75 and 25 repeating units (RU), respectively, typically observed in 1418 *ΔtolR* GMMA [30], changed into one main population (consisting of 45 RU) after fHbp expression (Table 1). LPS content of 1418 *ΔtolR ΔwbaP* GMMA over total protein did not change after fHbp expression.

### 2.2. fHbp Heterologously Expressed Is in the Lumen of GMMA

Once demonstrated that the heterologous antigen fHbp was expressed in *S.* Typhimurium GMMA, the next question was to further investigate its location. To do this we developed a panel of analytical techniques aimed at not altering the sample structure. 

**Figure 2:**
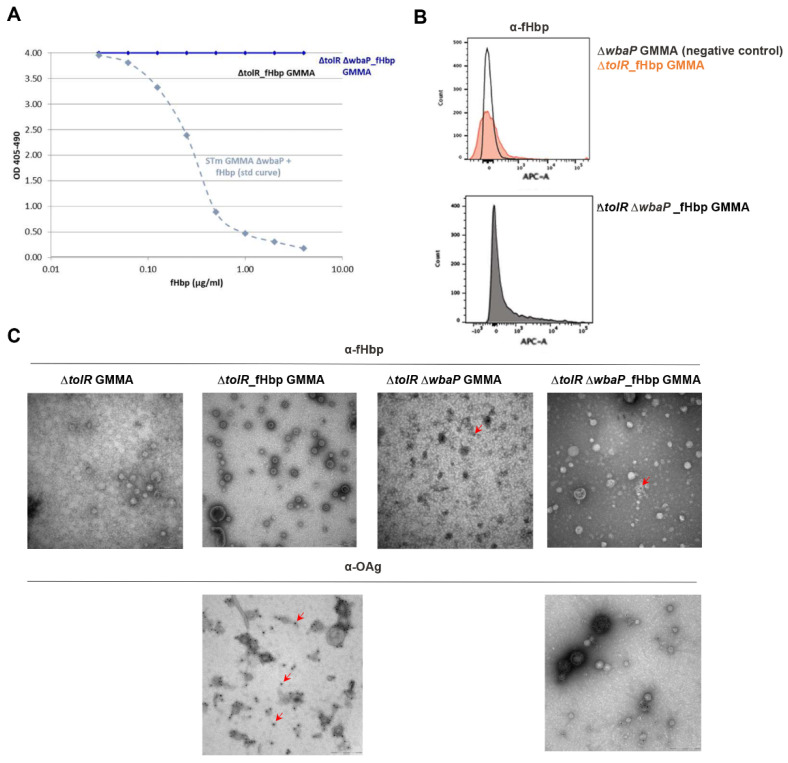
fHbp heterologously expressed is localized in the lumen of *S.* Typhimurium GMMA. (**A**) Competitive ELISA on the two *S.* Tyhpimurium GMMA samples overexpressing fHbp (OAg+ and OAg−). (**B**) FACS analysis performed on OAg+ and OAg− *S.* Typhimurium GMMA overexpressing fHbp, and on OAg− *S.* Typhimurium GMMA without expression of fHbp as negative control. (**C**) Surface analysis by TEM of OAg+ and OAg− *S.* Typhimurium GMMA with and without fHbp expression: immunogold labelling with anti-fHbp and anti-OAg specific antibodies. Red arrows indicate few examples of labelled particles.

We set up an anti-fHbp specific competitive ELISA able to detect the protein in the presence of GMMA, as shown by the standard curve built by serially diluting a known amount of fHbp physically mixed with GMMA derived from a *S.* Typhimurium 1418 *ΔtolR ΔwbaP* strain (Figure 2A). When diluting GMMA from *S.* Typhimurium 1418 *ΔtolR*_fHbp and 1418 *ΔtolR ΔwbaP*_fHbp strains, no detection of the antigen was observed by the assay, indicating that the foreign protein was not surface exposed. 

To confirm this first observation, we directly analyzed the different GMMA constructs by FACS with an anti-fHbp monoclonal antibody. As shown in Figure 2B, no positive staining was observed in the 1418 *ΔtolR*_fHbp GMMA nor in the 1418 *ΔtolR ΔwbaP*_fHbp GMMA.

Immunogold labeling by TEM additionally confirmed no presence of fHbp on the surface of both GMMA constructs (Figure 2C). We observed very few gold particles stained by the anti-fHbp antibody in the 1418 *ΔtolR ΔwbaP*_fHbp GMMA sample, but since this was also seen with the corresponding fHbp-negative 1418 *ΔtolR ΔwbaP* GMMA, we concluded it was related to a nonspecific binding (Figure 2C). 

Taken together these results demonstrated that fHbp heterologously expressed in *S.* Typhimurium GMMA was not surface exposed but located in the lumen of GMMA.

### 2.3. S. Typhimurium GMMA Expressing no Surface Exposed fHbp Induced Marginal Antibody Response in Mice

Having demonstrated that fHbp heterologously expressed in *S.* Typhimurium GMMA is not surface exposed, we next investigated whether this antigen was capable of inducing a specific antibody response. 

**Figure 3:**
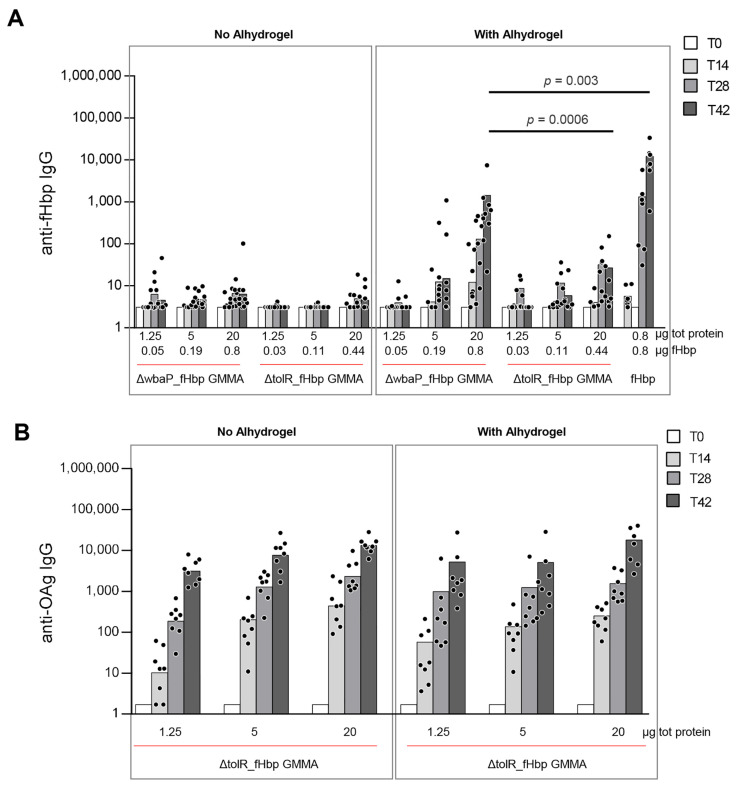
*S.* Typhimurium GMMA expressing no surface exposed fHbp tested in mice at different doses and with or without Alhydrogel. (**A**) anti-fHbp IgG response induced in mice by *ΔtolR ΔwbaP*_fHbp and *ΔtolR*_fHbp GMMA at pre-vaccination (T0) and at 14, 28 and 42 days post-vaccination. (**B**) anti-OAg IgG response induced in mice by *ΔtolR*_fHbp GMMA at pre-vaccination (T0) and at 14, 28 and 42 days post vaccination. Bars represent IgG geometric means and dots are individual antibody levels.

To this aim, mice were s.c. immunized twice with different doses of 1418 *ΔtolR*_fHbp or 1418 *ΔtolR ΔwbaP*_fHbp GMMA, in the presence or not of Alhydrogel as an adjuvant. As a control, we used recombinant fHbp, only formulated with Alhydrogel, as the presence of the adjuvant is necessary to induce a good immune response at the dose tested. An anti-fHbp specific IgG response was measured at 2-week intervals after the first and second injections. No anti-fHbp IgG antibodies were detected when GMMA were immunized in the absence of Alhydrogel. Only the highest dose of 1418 *ΔtolR ΔwbaP*_fHbp GMMA (20 µg total protein dose, corresponding to 0.8 µg of fHbp) was able to induce an anti-fHbp IgG response in the presence of Alhydrogel, significantly higher at day 42 compared to OAg+ GMMA expressing fHbp. Nevertheless, the anti-fHbp response induced was significantly lower compared to the one induced by the same dose of recombinant fHbp alone administered with Alhydrogel (Figure 3A). 

The OAg positive GMMA (1418 *ΔtolR*_fHbp) were able to induce an anti-OAg specific IgG response despite the expression of the heterologous protein. Alhydrogel did not increase an anti-OAg IgG response, as previously verified for *S.* Typhimurium GMMA [31] (Figure 3B).

### 2.4. Investigating S. Typhimurium 1418 ΔtolR ΔwbaP_fHbp GMMA Stability on Alhydrogel

No surface exposure of fHbp in *S.* Typhimurium GMMA seemed to have a negative impact on the antigen-specific IgG response induced in mice. A marginal anti-fHbp IgG response was detected only after immunization with 1418 *ΔtolR ΔwbaP*_fHbp GMMA together with Alhydrogel. Some possible explanations for the observed antibody response could be (1) the higher content of fHbp in 1418 *ΔtolR ΔwbaP*_fHbp GMMA compared to 1418 *ΔtolR*_fHbp GMMA and relative different fHbp to total protein ratios; (2) the absence of OAg on GMMA positively impacting an anti-fHbp response or (3) the lower stability of 1418 *ΔtolR ΔwbaP*_fHbp GMMA when compared to the *ΔtolR* construct. 

**Figure 4:**
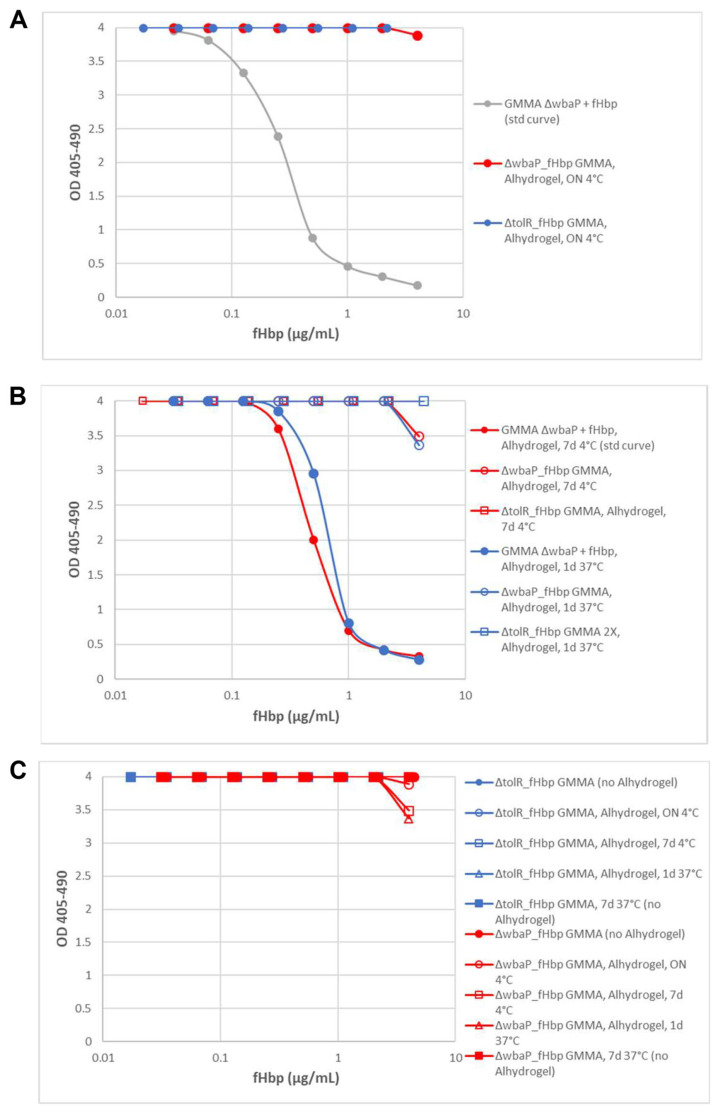
Stability assessment of 1418 *ΔtolR ΔwbaP*_fHbp and 1418 *ΔtolR*_fHbp GMMA on Alhydrogel. (**A**) FAcE assay performed on two freshly prepared Alhydrogel-formulated 1418 *ΔtolR ΔwbaP*_fHbp and 1418 *ΔtolR*_fHbp GMMA. Standard curve built by 1418 *ΔtolR ΔwbaP* GMMA physically mixed with recombinant fHbp. (**B**) FAcE assay performed on Alhydrogel-formulated 1418 *ΔtolR ΔwbaP*_fHbp and 1418 *ΔtolR*_fHbp GMMA incubated for 7 days at 4 °C and 1 day at 37 °C. Physical mixture of 1418 ΔtolR ΔwbaP GMMA and fHbp have been treated and incubated under the same conditions and used as standard curves. (**C**) Summary of the conditions used to assess stability of 1418 *ΔtolR ΔwbaP*_fHbp and 1418 *ΔtolR*_fHbp GMMA by FAcE and competitive ELISA.

FAcE assay conducted on freshly formulated 1418 *ΔtolR ΔwbaP*_fHbp and *ΔtolR*_fHbp GMMA (ON incubation on Alhydrogel at 4 °C) showed that formulation seems to partially affect membrane stability of OAg-negative GMMA only. The point of the curve at the highest concentration had OD slightly lower compared to the signal from total monoclonal binding, meaning that a certain amount of fHbp, corresponding to 0.08% of total protein content and to 1.9% of total fHbp (Figure 4A), had been released from the GMMA and bound by the anti-fHbp mAb. To further analyze the stability profile of the two formulated GMMA, we investigated two additional conditions: (1) 4 °C for 7 days and (2) 37 °C for 1 day. To be sure that conditions tested did not impact protein antigen stability, positive controls, consisting of formulated physical mixtures of 1418 *ΔtolR ΔwbaP* GMMA and fHbp, were subjected to the same treatments. These physical mixtures were also used as a standard curve for fHbp quantification in the samples treated under the same corresponding conditions. After 7 days of incubation at 4 °C, the 1418 *ΔtolR*_fHbp GMMA remained stable, while % of the fHbp detected by FAcE in the 1418 *ΔtolR ΔwbaP*_fHbp GMMA increased to 6.9% (Figure 4B). Treatment at 37 °C for 1 day also seemed to increase the level of disrupted vesicles in formulated OAg-negative GMMA, which rose to 10.9% detectable fHbp (Figure 4B). Formulated OAg-positive GMMA, instead, did not show loss of integrity, suggesting a higher stability profile. However, since the percentage of fHbp present in OAg-positive GMMA is almost half that of OAg-negative, we doubled the amount of 1418 *ΔtolR*_fHbp GMMA in the formulations to see if the differences found were just a matter of detectability and not linked to differences in stability. Again, no fHbp signal was detected (Figure 4B), confirming the major stability of OAg-positive compared to OAg-negative GMMA on Alhydrogel. 

Stability studies on OAg− and OAg+ GMMA expressing fHbp, based on dls measurements and HPLC-SEC/MALS analysis, showed that these particles, in absence of Alhydrogel, are stable up to 1 week at temperatures ranging from 4 to 40 °C (data not shown). This was confirmed in a competitive-ELISA performed on non-formulated 1418 *ΔtolR ΔwbaP*_fHbp and 1418 *ΔtolR*_fHbp GMMA incubated at 37 °C for a week, in which no presence of fHbp was revealed (Figure 4C). This confirms that Alhydrogel might play a role in destabilizing GMMA outer membranes, in particular OAg-negative GMMA, that seem to be intrinsically less stable (Table 2).

### 2.5. Exposure of fHbp on GMMA Surface Increased Antigen-Specific Immune Response

Our data demonstrated that the *S.* Typhimurium 1418 *ΔtolR ΔwbaP*_fHbp GMMA were less stable when administered with Alhydrogel, allowing a marginal anti-fHbp antibody response in mice. 

**Figure 5:**
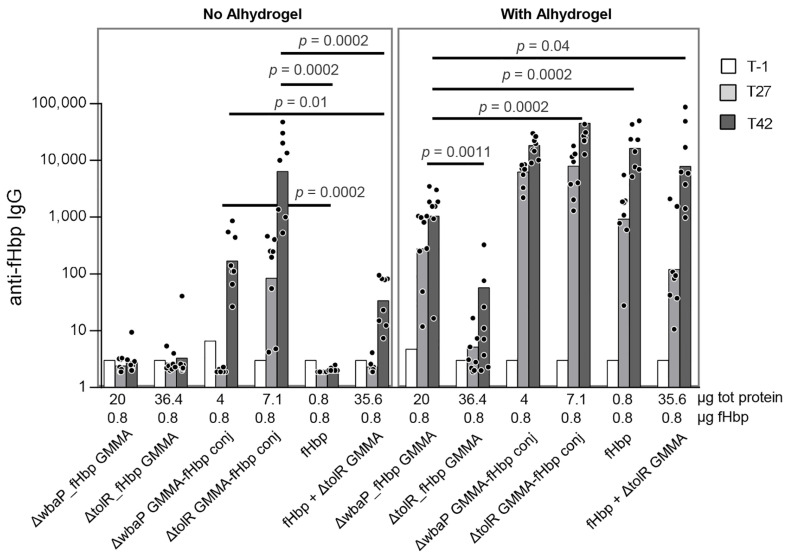
*S.* Typhimurium GMMA expressing no surface exposed fHbp tested in mice in comparison to fHbp chemically conjugated to GMMA surface. Anti-fHbp IgG response induced in mice by *ΔtolR ΔwbaP*_fHbp and *ΔtolR*_fHbp GMMA and *ΔtolR* or *ΔwbaP* GMMA chemically conjugated to fHbp at pre-vaccination (T-1) and at 27 and 42 days post-vaccination. Bars represent IgG geometric means and dots are individual antibody levels.

In order to validate the importance of antigen surface exposure for inducing an immune response, as well as to exclude any possible masking effect of OAg versus the heterologous protein, we chemically conjugated the fHbp to the 1418 *ΔtolR* GMMA and 1418 *ΔtolR ΔwbaP* GMMA, as a quick way to deliver the antigen on the GMMA surface. Mice were s.c. immunized twice with one dose of the fHbp (0.8 µg dose) chemically linked to the 1418 *ΔtolR* and 1418 *ΔtolR ΔwbaP* GMMA or expressed in both the 1418 *ΔtolR* and 1418 *ΔtolR ΔwbaP* GMMA, in the presence or not of Alhydrogel as an adjuvant. Mice groups receiving the same dose of the fHbp alone or physically mixed with the 1418 *ΔtolR* GMMA were also included as controls. Anti-fHbp specific antibody response was measured after the first and second injections. GMMA constructs with the fHbp not surface exposed (1418 *ΔtolR ΔwbaP*_fHbp and 1418 *ΔtolR*_fHbp GMMA) were not able to induce a significant response in mice in the absence of Alhydrogel as expected, but differently from the 1418 *ΔtolR* and 1418 *ΔtolR ΔwbaP* GMMA conjugates, where fHbp was linked on GMMA surface, which induced a higher antibody response compared to the antigen alone or physically mixed with the GMMA (Figure 5). At the same fHbp dose, also in this animal study, only the 1418 *ΔtolR ΔwbaP*_fHbp GMMA construct was able to induce an anti-fHbp IgG response significantly higher than the 1418 *ΔtolR*_fHbp GMMA when formulated with Alhydrogel (*p* = 0.0011, by Mann-Whitney test two-tailed comparison at day 42), but still significantly lower than the fHbp alone or physically mixed with the GMMA, and even lower compared to the 1418 *ΔtolR* GMMA-fHbp conjugate (*p* = 0.0002 by Mann-Whitney two-tailed test at day 42) (Figure 5). The presence of OAg did not interfere with an anti-fHbp IgG response, as shown by comparing the anti-fHbp IgG response elicited by OAg+ (1418 *ΔtolR* GMMA-fHbp conjugate) and OAg− conjugates (1418 *ΔtolR ΔwbaP* GMMA-fHbp conjugate). We also confirmed the ability of GMMA conjugates to induce an anti-OAg IgG response in the presence of fHbp by anti-OAg IgG ELISA (Supplementary Figure S1).

An additional study was performed to assess the ability of fHbp-GMMA conjugates to induce bactericidal anti-fHbp antibodies, in comparison to fHbp alone or as a physical mixture with GMMA, at the same relative amount of fHbp (0.75 µg) and GMMA (1.75 µg), in the presence of Alhydrogel. Expected anti-fHbp and anti-OAg antibody responses were measured by ELISA (Supplementary Figure S2). Bactericidal activity of the antibodies induced was assessed against homologous *Neisseria meningitidis* serogroup B (MenB) strains, carrying the same variant of fHbp, and *S.* Typhimurium strain. We observed that antibodies from mice immunized with the fHbp chemically conjugated to the GMMA surface were able to kill the homologous strains, even at earlier timepoints (14 days after the first injection) compared to those elicited by the fHbp alone or as physically mixed with the GMMA (Table 3). Bactericidal titers against *S.* Typhimurium strain confirmed that the presence of fHbp on GMMA did not interfere with the immunogenicity of GMMA.

## 3. Discussion

Heterologous antigen expression in membrane vesicles has opened the way to OMV-based multivalent vaccines. Heterologous antigens can be presented on the OMV with or without surface exposure, attached to the vesicle or simply mixed to them [32]. Hence, various possibilities of antigen location exist, and at the moment it is not possible to predict the optimal design for a multivalent OMV-based vaccine. 

It has been previously reported that antigen-specific humoral immunity can increase significantly when antigens are exported either to the carrier surface or extracellularly into the surrounding milieu, rather than remaining in the cytoplasm [33,34,35]. While surface exposed antigens are accessible for antigen-specific B cell binding, the inside of the OMV is hindered from their recognition, and this may play a role in the lower/minimal antibody titer induced by OMV-associated internal antigens. To this end, it has been previously shown how luminal antigens may be skewed towards cytotoxic T-cell responses [34]. Many groups have expressed heterologous antigens either in the lumen or on the surface of OMVs and have speculated on the need or not to have the antigen exposed on the OMVs surface. However, only few studies have reported a direct comparison between OMVs presenting the same antigen on the surface or in the lumen of the vesicles. Schild et al. have investigated an intranasal immunization model using *V. cholerae* OMVs loaded with *E. coli* alkaline phosphatase PhoA and observed a small, but significant, immune response against PhoA. The relatively low immune response to PhoA might be due to the location of the enzyme in the lumen of the OMV as opposed to the surface [23]. In another study, proteins from Group A *Streptococcus* (GAS) (i.e., Slo, SpyCEP, SpyAD) and Group B *Streptococcus* (GBS) (i.e., SAM_1372) were fused to the OmpA leader sequence for secretion and incorporated as native proteins into the lumen of *E. coli* OMVs. Following immunization in mice, all the OMVs induced high functional antibody titers against the recombinant proteins. Furthermore, immunization with Slo-OMV and SpyCEP-OMV protected mice against GAS lethal challenge [24]. 

Similarly, mice immunized intranasally with *S.* Typhimurium OMVs, engineered to contain the pneumococcal protein PspA in the OMV lumen, developed serum antibody responses against PspA, *Salmonella* LPS and outer membrane proteins, while no detectable responses were developed in mice immunized with an equivalent dose of recombinant PspA alone [25]. Mucosal IgA responses were developed against the *Salmonella* antigen components, while the response to PspA was less apparent in most of the immunized mice. Mice immunized with the recombinant OMVs were protected against challenge with *Streptococcus pneumoniae*. However, further studies suggested to assess whether the anti-PspA immune response could have been enhanced by localizing the antigen at the surface of the OMVs [25]. Salverda and collaborators have also demonstrated that the expression of the Borrelial surface-exposed lipoprotein OspA on the surface of *Neisseria meningitidis* OMVs allowed the induction of a strong anti-OspA antibody response compared to the construct with luminal expression of OspA where no antigen-specific antibody response was observed [36]. The presentation of a luminal protein to naïve B cells requires that the integrity of OMVs is partially compromised in order to expose the protein to B cell receptors. As also proposed by Fantappiè and collaborators, a fraction of OMVs can be degraded at the injection site allowing antigen presentation to naïve B cells. Therefore, the ratio between intact and degraded OMVs at the site of injection is important for the generation of a sufficiently large population of antigen-specific T- and B-cells and, ultimately, for a good antibody response. 

In our study, we focused on investigating co-expression of antigens targeting *Neisseria* and *Salmonella* disease, with the ultimate goal of developing a vaccine that could be useful for Africa, where *Neisseria meningitidis* and nontyphoidal *Salmonella* are both prevalent [14,27,28]. We heterologously expressed *Neisseria meningitidis* fHbp into *Salmonella* Typhimurium OAg+and OAg− hyperblebbing strains. With OAg being a key target antigen for a Salmonella immune response, we were interested in investigating whether an anti-OAg antibody response could interfere with and dominate the immune response versus a heterologous protein antigen, or vice versa. 

The heterologous antigen fHbp expressed in *Salmonella* GMMA was internalized in the lumen, as we demonstrated by a panel of analytical and characterization methods. When the fHbp was not presented on the GMMA surface, this had a negative impact on the anti-fHbp IgG response induced. The only construct able to give a significant response, still lower than recombinant fHbp, was the OAg− GMMA (ΔtolR ΔwbaP_fHbp), only when adjuvanted with Alhydrogel. We found this to be possibly explained by a partial instability of OAg-GMMA on Alhydrogel, as demonstrated by a competitive ELISA run on formulated material. 

We next demonstrated the essentiality of the fHbp surface exposure on the GMMA to induce a strong antigen-specific antibody response by using chemical conjugation [26]. Interestingly, the IgG level induced by the fHbp-conjugated GMMA was even higher than the response induced by the fHbp alone or in a physical mixture with the GMMA. Importantly, we have also confirmed that when the fHbp is surface exposed, the presence of OAg on the GMMA does not interfere with the anti-fHbp IgG response, or vice versa. These data support the idea that our OMV technology can be used to develop a bivalent vaccine, as we have here demonstrated using *N. meningitidis* and *Salmonella* as model pathogens targeting a protein and a polysaccharide antigen, respectively.

Overall, our results clearly confirm that surface exposure of fHbp on GMMA is essential for eliciting a high humoral response. Partial instability of the OAg-GMMA, expressing fHbp in the lumen, was found to be likely responsible for the low anti-fHbp IgG response induced by this construct. These results reinforce the idea that degradation of GMMA/OMVs at the site of injection could explain the presentation of luminal proteins to naïve B cells, as previously proposed [24]. In light of immunization, the formulation and nature of GMMA are important factors to be considered as they can impact vesicles’ integrity and the overall extent of immune response induced. It would now be interesting to extend the work done here using fHbp to other antigens, to see if the nature of the heterologous antigen displayed on GMMA can influence the immunogenicity profile.

## 4. Materials and Methods

### 4.1. Bacterial Strains and Generation of Mutants

*Salmonella enterica* serovar Typhimurium isolate SGSC1418 (STm 1418, LT-2 collection, University of Calgary) was used as parent strain [37]. Mutants were generated by replacing the gene of interest with an antibiotic resistance cassette, by homologous recombination using lambda red recombineering system [38]. The *tolR* gene was replaced by kanamycin using the same primer sets previously reported [13,39], the *wbaP* gene was replaced by a chloramphenicol resistance cassette from plasmid pKD3 with a specific primer set (Forward primer: cgcaggctaatttatacaattattattcagtacttctcggtaagcGTGTAGGCTGGAGCTGCTTCG, Reverse primer: cttaatatgcctattttatttacattatgcacggtcagagggtgaCATATGAATATCCTCCTTAG). 

*Salmonella* strains were routinely grown in Luria-Bertani (LB) broth at 37 °C. When required, kanamycin or chloramphenicol were added to a final concentration of 30 µg/mL and 20 µg/mL, respectively.

### 4.2. Heterologous Antigen Expression and GMMA Preparation

The polymerase incomplete primer extension (PIPE) cloning method [40] was used to insert fHbp (variant 1-ID5 from *Neisseria meningitidis* serogroup A strain) [41] into a pGEX-derived plasmid, carrying the sequence encoding the leader peptide for secretion of *E. coli* OmpA. The *Escherichia coli* HK-100 strain was used for cloning and plasmid generation and was grown in Luria-Bertani (LB) broth at 37 °C. Ampicillin was used at the final concentration of 100 µg/mL. *Salmonella* mutant strains carrying the plasmid encoding for fHbp were grown in LB at 37 °C. Heterologous protein expression was induced by adding 1 mM IPTG when the cultures reached 0.5 OD/mL. After 24 h of protein induction, culture supernatants were collected and the GMMA purified as previously described [13].

### 4.3. GMMA Characterization

Total protein content was estimated by micro BCA. The content of the O-antigen (OAg) portion of *Salmonella* lipopolysaccharide (LPS) was quantified by High-Performance Anion-Exchange Chromatography coupled to Pulsed Amperometric Detector (HPAEC-PAD) as previously described [30,42], after performing acid hydrolysis directly on the GMMA. The GMMA components did not interfere in the quantification of the OAg sugar monomers. The amount of core reducing end KDO (2-keto-3-deoxy-octonate) was assumed equal to the amount of lipid A and quantified by semicarbazide/High Performance Liquid Chromatography-Size Exclusion Chromatography (HPLC-SEC) method after sugar extraction [42]. 

OAg chains were extracted, purified and characterized by HPLC-SEC analysis and HPAEC-PAD to determine the sugar length and number of OAg repeating units as previously described [30]. 

The GMMA particle size was estimated by HPLC-SEC/Multi-Angle Light Scattering (HPLC-SEC/MALS) and dynamic light scattering (dls) as previously reported [43]. 

FASP/H3 Mass Spectrometry [44,45,46] was used to quantify meningococcal fHbp expressed in the GMMA preparations.

### 4.4. SDS-Page and Western-Blotting Analysis 

Protein pattern profile and fHbp protein expression were analyzed by SDS-PAGE [30] and Western-blotting analysis. The gel was trans-blotted to a nitrocellulose membrane (iBlot, Thermo Fisher Scientific, USA). The membrane was blocked with 3% BSA-PBS Tween-20 0.05% and immuno-stained with anti-fHbp mice polyclonal serum (2 h at room temperature, RT) diluted at 1:2000, followed by anti-mouse IgG (1 h at RT) conjugated to alkaline phosphatase and developed with alkaline phosphatase substrate kit (Merck, Darmstadt, Germany). Both primary and secondary antibodies were diluted in 0.1% BSA-PBS Tween-20 0.05%.

### 4.5. Flow Cytometry Analysis of GMMA Samples

The GMMA samples were analyzed by FACS following instructions as previously reported [47]. The GMMA samples at the concentration of 40 µg/mL (total protein) in PBS BSA 1% were stained by incubation with the anti-fHbp monoclonal antibody (mAb) (Jar4, from NIBSC, London, UK) diluted at 1:50 for 1 h at 4 °C. One wash was performed by ultracentrifugation of the GMMA samples (110,000 rpm, 4 °C, 30 min) and resuspension in PBS BSA 1%, followed by incubation with a secondary antibody Alexa Fluor 647 F(ab’)2 fragment Goat Anti-Mouse IgG (Thermo Fisher Scientific, Waltham, MA, USA) diluted at 1:200 for 1 h on ice. The washing step by ultracentrifugation was repeated and samples resuspended in PBS and analyzed for GMMA-bound fluorescence using a FACS Fortessa flow cytometer (BD Biosciences, San Jose, CA, USA). Flow cytometry data were processed using FlowJo software (BD Biosciences, San Jose, CA, USA). 

### 4.6. TEM and Immunogold Labeling 

The GMMA were adsorbed to Formvar/carbon-coated grids, negatively stained with uranyl acetate, as described previously [48], and subsequently observed with a Tecnai G2 Spirit transmission electron microscope (FEI, Eindhoven, The Netherlands) operating at 80 kV. Electron micrographs were recorded at a nominal magnification of X87,000. GMMA diameters were manually measured in comparison with the scale bar. Immunogold labeling was obtained by staining with anti-fHbp or anti-OAg mice polyclonal sera. 

For analysis by immunogold staining, a 5 µL aliquot of GMMA with a final concentration of 100 µg/mL were adsorbed to 300-mesh nickel grids, blocked in PBS with 0.5% bovine serum albumin (BSA) and incubated with primary anti-fHbp polyclonal serum (diluted 1: 400 in PBS with 1% BSA) or primary anti-OAg mAb (AbCAM, diluted 1:1000) for 1 h. Grids were washed several times in PBS with 1% bovine serum albumin and incubated with gold-labeled anti-mouse secondary antibody (diluted 1: 40 in PBS with 1% bovine serum albumin) for 1 h. After several washes with distilled water the grids were negatively stained and analyzed using a TEM FEI Tecnai G2 spirit microscope.

### 4.7. Formulated Alhydrogel Competitive ELISA (FAcE) and Competitive ELISA

FAcE and competitive ELISA assays were performed as previously described [49]. For the FAcE assay, a standard curve was prepared by 8 sequential 2-fold dilution steps of Alhydrogel formulated *S.* Typhimurium GMMA physically mixed with pure recombinant fHbp variant 1, starting from a known concentration of 4 µg/mL fHbp and 100 µg/mL GMMA. Formulated *S.* Typhimurium GMMA overexpressing fHbp (1418 *ΔtolR*_fHbp and 1418 *ΔtolR ΔwbaP*_fHbp) were tested as undiluted (starting concentration in total protein of 100 µg/mL) and then in 7 sequential 2-fold dilutions. Standard curve and test samples were diluted in Alhydrogel diluent (Alhydrogel 0.7 mg/mL in saline). Anti-fHbp mAb was diluted in PBS with 1% BSA and 0.5% Tween-20 and then added to the diluted samples in a ratio 1:1 (mAb final dilution 1:500). All mixtures were pre-incubated overnight (ON) at 4 °C shaking. Each assay was run in duplicates in 2 independent plates (Nunc round bottom Maxisorp ELISA plates). Plates were coated ON at 4 °C with recombinant fHbp at 1 µg/mL in carbonate buffer pH 9.6. The coating solution was removed, and plates blocked with 5% fat-free milk dissolved in PBS buffer. After 1 h incubation at 25 °C, plates were washed three times with washing buffer (PBS with 0.05% Tween-20). The pre-incubated mixtures of samples/standard curve with mAb were added to each ELISA plate, and the plates were incubated for 2 h at RT (about 21 °C) in a plate shaker (MixMate from Eppendorf AG, Hamburg, Germany) set at 600 rpm. Plates washing step was then repeated and a secondary antibody anti-mouse IgG conjugated to alkaline phosphatase (A3438, Merck, Darmstadt, Germany) was added to the plates and incubated for 1 h at 25 °C. After three more washes in a washing buffer, 100 µL of p-nitrophenyl phosphate substrate solution (N2770, Merck, Darmstadt, Germany) were added and plates were incubated for 1 h at 25 °C. Absorbance was read at 405 nm and 490 nm and the difference between them (OD405nm-OD409nm) was measured. The standard curve was fitted with a four-parameter logistic curve model using GraphPad Prism 6 software (GraphPad Software, La Jolla, CA, USA), and the amount of fHbp in the test samples was calculated by nonlinear regression analysis. 

Competitive ELISA was performed following the same conditions, but the samples were not Alhydrogel-formulated, hence dilutions were performed in ELISA Sample Dilution Buffer (PBS with 0.1% BSA and 0.5% Tween-20).

### 4.8. Formulation Procedure for GMMA Stability Assessment 

*S.* Typhimurium GMMA overexpressing fHbp (1418 *ΔtolR*_fHbp and 1418 *ΔtolR ΔwbaP*_fHbp) were diluted in saline and formulated in complete sterility with Alhydrogel, to have 40 (or 80) μg protein content in a total final volume of 400 μL and with Al^3+^ concentration of 0.7 mg/mL. As a positive control, physical mixtures were prepared with 40 μg of the 1418 *ΔtolR ΔwbaP* GMMA and 1.6 μg of the fHbp recombinant protein (corresponding to 4% of GMMA total protein content, thus the amount of fHbp present in 1418 *ΔtolR ΔwbaP*_fHbp GMMA). After formulation, all samples were allowed to stand at 4 °C ON on a rotator. To check the GMMA stability over time, samples were stored at 4 °C or 37 °C for different time periods.

### 4.9. fHbp GMMA Conjugate Preparation

The fHbp (from group variant 1) was linked to both OAg+ and OAg− *S.* Typhimurium GMMA surface proteins either using the bis(sulfosuccinimidyl)suberate (BS3) linker or the N-acetyl-DL-homocysteine thiolactone (SH-maleimido) linker as previously described [26]. Western blot characterization confirmed conjugate formation and no unconjugated protein was detected by HPLC-SEC. Amino acid analysis as reported before [26] was done to quantify the exact amount of the fHbp linked to the GMMA and to compare all constructs at the same fHbp dose. Conjugates obtained via BS3 chemistry resulted in having 11.2% fHbp/total proteins for OAg+ conjugate, and 19.8% fHbp/total proteins for OAg− conjugate. In the case of fHbp-GMMA conjugates obtained by SH-maleimido chemistry, a 30% fHbp/total proteins ratio was obtained. 

### 4.10. Mouse Immunogenicity Studies

All animal sera used in this study derived from immunization experiments performed at Toscana Life Sciences Animal Facility (Siena, Italy), in compliance with the relevant guidelines (Italian D. Lgs. n. 26/14 and European directive 2010/63/UE) and the institutional policies of GSK. The animal protocols were approved by the Animal Welfare Body of Toscana Life Sciences and the Italian Ministry of Health (Approval number 201309 and 479/2017-PR). CD1 5-week-old outbred female mice were immunized subcutaneously (s.c.) at days 0 and 28. Antigens were diluted in saline or diluted in saline with Alhydrogel (0.7 mg/mL, Al^3+^) the day before each vaccination. Sera were collected at day 0 (pre-bleed) and at days 14 and/or 28. The bleed out was performed on day 42. Pre-bleed were pooled, while bleeds at day 14, 28 and 42 were kept as individual sera. 

Anti-antigen-specific IgG levels were measured at days 0, 14, 28 and 42 by Enzyme-Linked Immunosorbent Assay (ELISA) [50]. Purified fHbp v1 and OAg from *S.* Typhimurium were used for ELISA plate-coating at the concentration of 1 µg/mL or 5 µg/mL, respectively, in carbonate buffer pH 9.6. ELISA units were expressed relative to a mouse antigen-specific antibody standard serum curve, with the best 5 parameter fit determined by a modified Hill plot. One ELISA unit is defined as the reciprocal of the dilution of the standard serum that gives an absorbance value equal to 1 in this assay. Each mouse serum was run in triplicate.

Serum Bactericidal Activity (SBA) against meningococcal B and *S.* Typhimurium strains was tested using baby rabbit complement as previously described [14,31,51]. 

### 4.11. Statistical Analysis

Datasets were analyzed using a two-tailed nonparametric Mann–Whitney test with Prism (GraphPad Software, San Diego, CA, USA). *p*-values lower than 0.05 were considered statistically significant, and *p*-values were rounded to the nearest larger number.

## Figures and Tables

**Table 1:** Characterization of GMMA from *S.* Typhimurium 1418 *ΔtolR* and *ΔtolRΔwbaP* mutated strains with and without fHbp expression: particle size, OAg, protein and LPS content. n/a: not applicable.

GMMA	dls Z Average d_(PdI) nm	MALS (2 × Rw nm)	Molar Ratio to Rha			OAg/Protein *w*/*w* Ratio	Number OAg Repeats	LPS/ Protein (nmol/mg)	fHbp/Total Protein Expression (%)
			Gal	Glc	Man				
1418 Δ*tolR*_fHbp	111.4 (0.101)	70	0.99	0.55	0.82	0.9	45 (1 population)	141	2.2
1418 Δ*tolR*	91.4 (0.170)	71.5	1.05	0.96	0.98	0.66	75 and 25 (2 populations)	173	n/a
1418 Δ*tolR* Δ*wbaP*_fHbp	46.4 (0.149)	41.4	n/a	n/a	n/a	n/a	n/a	255	3.9
1418 Δ*tolR* Δ*wbaP*	57.6 (0.260)	52.6	n/a	n/a	n/a	n/a	n/a	272	n/a

**Table 2:** Summary of results obtained by FAcE or competitive ELISA for each condition tested on 1418 *ΔtolR ΔwbaP*_fHbp and 1418 *ΔtolR*_fHbp GMMA formulated on Alhydrogel, unless specified.

Tested Condition	% fHbp Detected/Total fHbp in GMMA	
	1418 *ΔtolR*_fHbp GMMA (OAg+)	1418 *ΔtolR ΔwbaP*_fHbp (OAg−)
No Alhydrogel	0.0%	0.0%
Freshly formulated GMMA (ON 4 °C)	0.0%	1.9%
7 d 4 °C	0.0%	6.9%
1 d 37 °C	0.0%	10.9%
7 d 37 °C No Alhydrogel	0.0%	0.0%

**Table 3:** SBA titers against MenB strains (H44/76 and M6190) and *S.* Typhimurium strain D23580.

Groups	SBA Titers Against MenB Strains			SBA Titers Against *S.* Typhimurium Strain
	H44/76	H44/76	M6190	D23580
	d14	d42	d42	d42
*ΔtolR* GMMA-fHbp conj	1000	>163,840	>163,840	66,002
fHbp + *ΔtolR* GMMA	40	>163,840	10,000	365,732
fHbp	<10	>163,840	2000	<100
*ΔtolR* GMMA	<10	<10	<10	73,310

## Data Availability

Data is contained within the article or supplementary material.

## Supplementary Materials

The following are available online at https://www.mdpi.com/article/10.3390/pathogens10060726/s1, Figure S1: Anti-OAg IgG response induced in mice by *S.* Typhimurium GMMA expressing no surface exposed fHbp or with fHbp chemically conjugated on their surface. Bars represent IgG geometric means and dots are individual antibody levels; Figure S2: Summary graphs of anti-fHbp (**A**) and anti-OAg (**B**) IgG geometric means (bars) and individual antibody levels (dots) induced in CD1 mice by *S.* Typhimurium ΔtolR GMMA chemically conjugated to fHbp, as well as by the physical mixture of STm GMMA with fHbp, or by fHbp alone, in the presence of Alhydrogel. Mice have been immunized twice subcutaneously at day 0 and 28, and were bled before first immunization and at days 14, 27 and 42 after first immunization.
