# Supplementary figures and images for: Neisseria meningitidis Factor H Binding Protein Surface Exposure on Salmonella Typhimurium GMMA Is Critical to Induce an Effective Immune Response against Both Diseases
Source: Pathogens. 2021 Jun 9;10(6):726. doi: 10.3390/pathogens10060726 (PMC8229706; doi:10.3390/pathogens10060726)

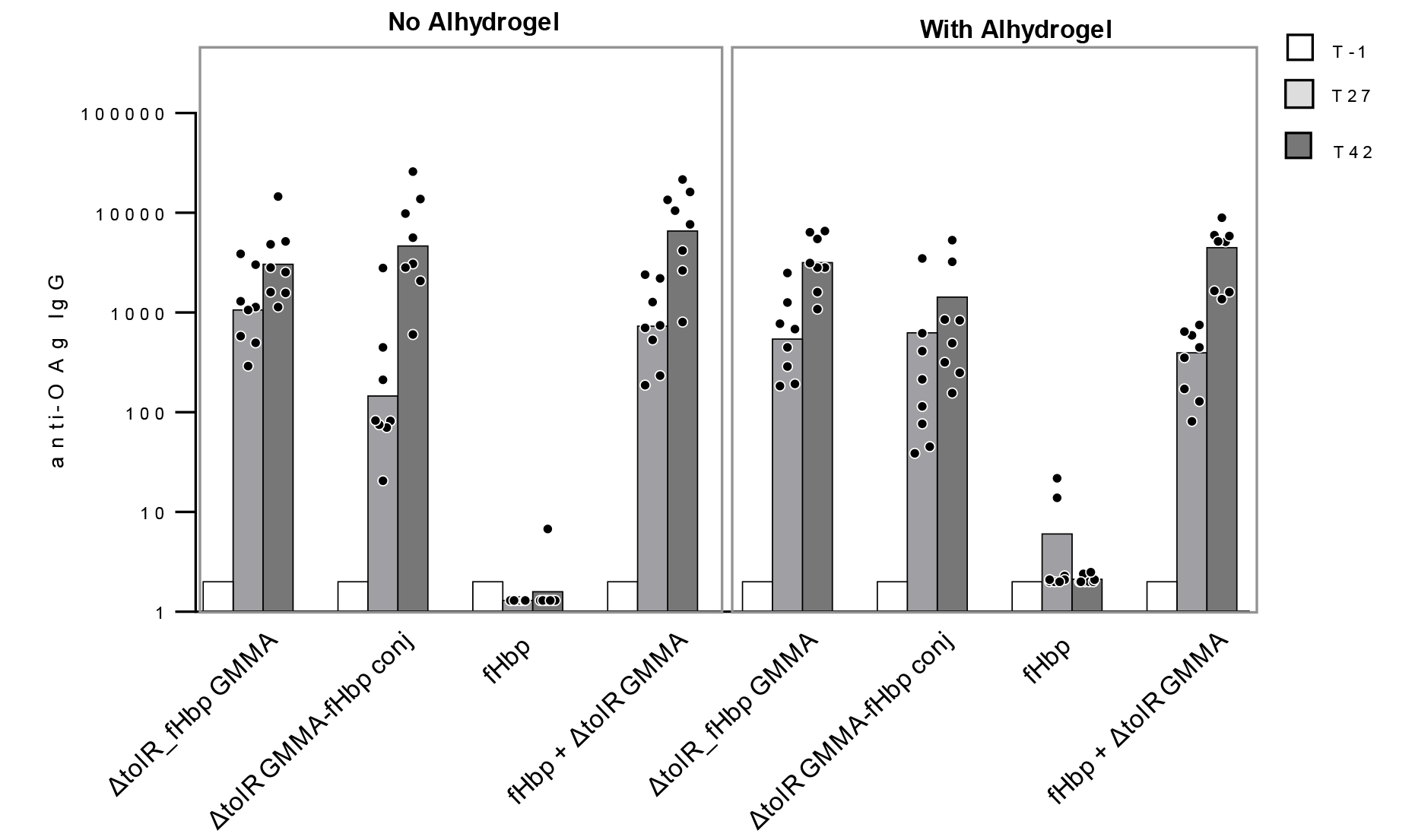

Supplement: Supplementary file 1 [file pathogens-10-00726-s001.zip › FigS1.tif]

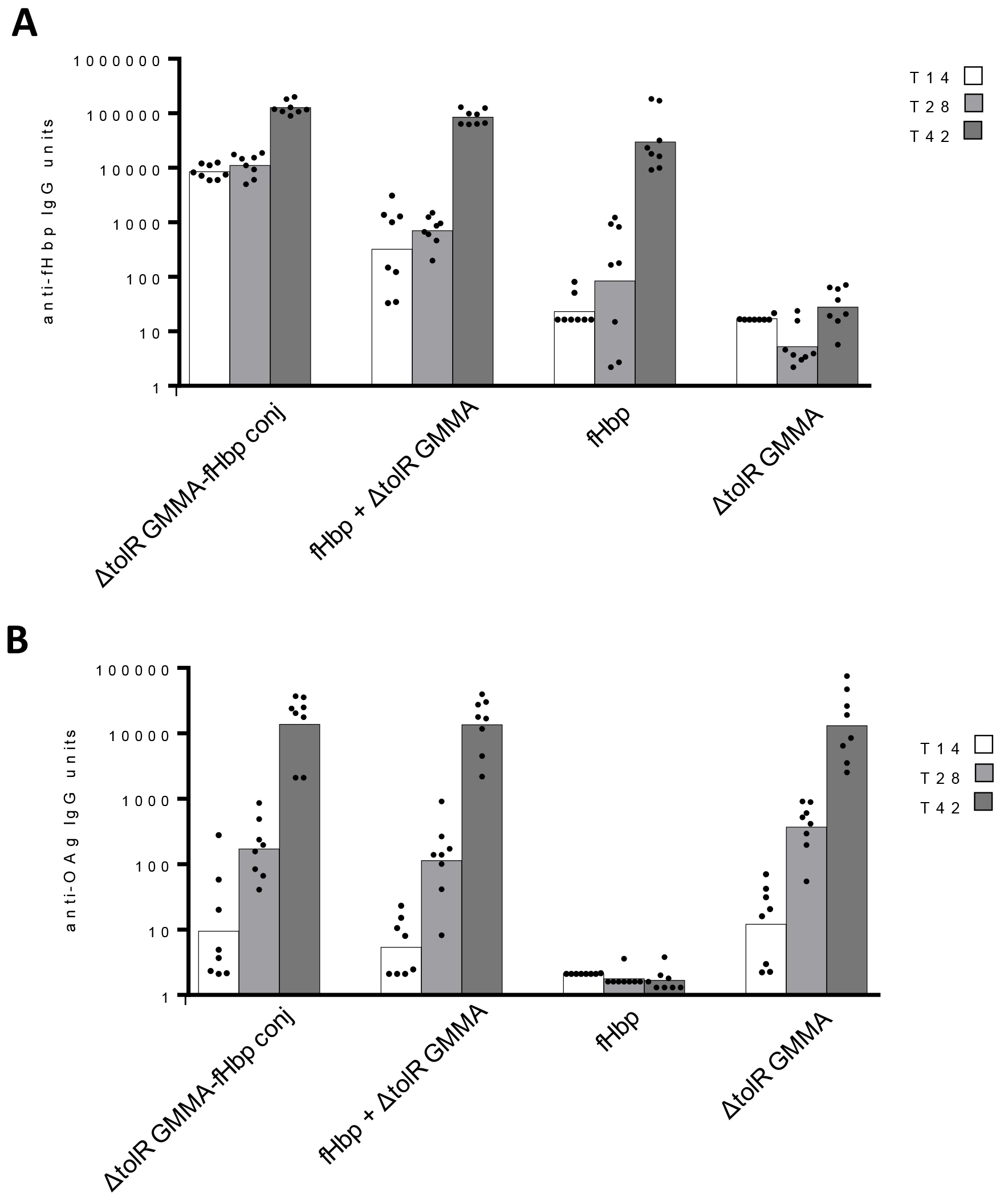

Supplement: Supplementary file 1 [file pathogens-10-00726-s001.zip › FigS2.tif]
